# Recognition of Damaged Arrow-Road Markings by Visible Light Camera Sensor Based on Convolutional Neural Network

**DOI:** 10.3390/s16122160

**Published:** 2016-12-16

**Authors:** Husan Vokhidov, Hyung Gil Hong, Jin Kyu Kang, Toan Minh Hoang, Kang Ryoung Park

**Affiliations:** Division of Electronics and Electrical Engineering, Dongguk University, 30 Pildong-ro 1-gil, Jung-gu, Seoul 100-715, Korea; vokhidovhusan@nate.com (H.V.); cagalli@naver.com (H.G.H.); kangjinkyu@dgu.edu (J.K.K.); hoangminhtoan@dongguk.edu (T.M.H.)

**Keywords:** arrow-road marking recognition, convolutional neural network, damaged arrow-road marking, visible light camera sensor, advanced driver assistance system (ADAS)

## Abstract

Automobile driver information as displayed on marked road signs indicates the state of the road, traffic conditions, proximity to schools, etc. These signs are important to insure the safety of the driver and pedestrians. They are also important input to the automated advanced driver assistance system (ADAS), installed in many automobiles. Over time, the arrow-road markings may be eroded or otherwise damaged by automobile contact, making it difficult for the driver to correctly identify the marking. Failure to properly identify an arrow-road marker creates a dangerous situation that may result in traffic accidents or pedestrian injury. Very little research exists that studies the problem of automated identification of damaged arrow-road marking painted on the road. In this study, we propose a method that uses a convolutional neural network (CNN) to recognize six types of arrow-road markings, possibly damaged, by visible light camera sensor. Experimental results with six databases of Road marking dataset, KITTI dataset, Málaga dataset 2009, Málaga urban dataset, Naver street view dataset, and Road/Lane detection evaluation 2013 dataset, show that our method outperforms conventional methods.

## 1. Introduction

Within the automotive industry, the advanced driver assistance system (ADAS) technology, in existence for many years, is now adding enhanced automated function that provides the driver and passengers with a higher level of safety and comfort relative to current ADASs.

An ADAS includes a variety of functions, e.g., automated cruise control, adaptive light control, parking assistance, collision avoidance, rear view, blind spot detection, driver drowsiness alert, global positioning system (GPS) navigation, lane departure warning, and intelligent speed control. Several of these technologies have been researched and are now implemented and integrated within many automobiles from a variety of manufacturers. In many cases, the results are an improved driving experience and better road safety. With an ADAS installed, a driver constantly receives visual images of the road and surroundings. The primary purpose of the road markings is to alert the driver or pedestrian relative to potential hazards and provide guidance, rules, or directions to drivers and pedestrians. In order to integrate the road marking recognition and reaction process within an ADAS, it is necessary to provide an automated visual recognition system for all road markings and implement timely responses to these markings, either by providing advice to the driver or, unilaterally, controlling the car to take the appropriate action.

Automatic recognition of road markings is a challenging problem to solve and integrate into an automotive vision system. Unlike traffic signs, road markings exist on road surfaces and they can be easily damaged. For example, directional arrows, numbers, and word messages are more likely to be damaged than traffic signs because the paint on the marking is eroded by vehicular traffic over time. A human brain is quite skilled at analyzing this information and can respond in a timely manner with an appropriate series of actions. However, in order to recognize damaged or indistinguishable road markings with a high degree of accuracy, a computer vision system must support very small response times and a high sensitivity to the field of vision. In the next Section, we provide detailed explanations of previous work in this research area.

## 2. Related Works

The problem of automated recognition of road markings has been studied by many researchers. Previous researchers used various image processing techniques to recognize road markings and signs [1,2,3,4]. For example, Foucher et al. [5] presented a method of detection and recognition of lane, crosswalks, arrows, and several related road markings, all painted on the road. They propose a road marking recognition method that consists of two steps: (1) extraction of marking elements; and (2) identification of connected components based on single pattern or repetitive rectangular patterns.

The template matching method was also used to implement road marking recognition. In [6], the maximally stable extremal regions (MSERs) were used to detect a region of interest (ROI) of road marking. In order to classify road markings, a histogram of oriented gradient (HOG) features and template matching methods was used. This method was proposed to detect and classify text and symbols; the results show a false positive rate of 0.9% and a true positive rate of 90.1%. Another template matching-based method was proposed in [7] for the recognition of road markings. Through the augmented transition network (ATN), the lanes are detected. Next, these lanes are used to establish the ROI that determines the boundaries in which the road markings, such as arrows, are located. Detected lanes that are valid are mostly used as a guide to detect markings. Ding et al. [8] presented a method for detection and identification of road markings. The researchers use HOG features and a support vector machine (SVM) to identify and classify five road markings. The method presented by Greenhalgh et al. [9] also used HOG features and a SVM for recognition of symbol-based road markings.

Text-based road-signs are recognized by an optical character recognition (OCR) method [1,10,11]. The system can recognize any random text word that might appear. In [12], a method was proposed that uses a Fourier descriptor and k-nearest neighbor (KNN) algorithm for recognition of road markings. In the fields of speed limit sign recognition, lane detection and traffic-sign detection and recognition, researchers have proposed techniques using an artificial neural network. One road-sign recognition algorithm is based on a neural network that uses color and shape information with back propagation, for the recognition of Japanese road signs [13]. In addition, the researchers used template matching and neural networks to recognize the road markings. The back propagation method is used as the learning method in a hierarchical neural network. The results show that the accuracy of the template matching algorithm remained lower than the accuracy of the neural network algorithm. Another approach to road-sign recognition is an earlier solution that uses artificial neural networks for the Bengali textual information box [14]; the results show a recognition accuracy of 91.48%.

While research of road-sign recognition using neural networks has been quite active, few research studies of road marking recognition using neural networks are available in the literature. One of the earliest methods for the recognition of arrow-marking was proposed by Baghdassarian et al. [15]. This research generated arrow-marking candidates through image binarization, and used a neural network with a chain code comparison for arrow classification. Another proposal uses a neural network to recognize road markings [16]. The researchers used the back propagation method as the learning method in a hierarchical neural network. They performed their experiments over six types of white road markings (turn left, turn right, turn left straight, turn right straight, straight, and crosswalk) and five orange road markings (30 km, 40 km, 50 km, 60 km, and U-turn ban). The experimental results showed that the average accuracy of recognition of white road markings was about 71.5%, while the average accuracy of recognition of orange markings was about 46%. Another research study proposed a method for detection and recognition of text and road marking [17]; this study extracts the shape-based feature vector from the candidates of road marking, and a neural network is used for classification of road marking. This approach shows a successful recognition rate of approximately 85% for arrows and 81% for the 19 dictionary words/text patterns. In [18], another machine learning-based method is proposed to detect and classify road markings. In this case, a binarized normed gradient (BING) and a principal component analysis (PCA) network with a SVM classifier were used for object detection and classification, respectively. In [19], the researchers used HOG features and a total error rate (TER)-based classifier for road marking classification; this resulted in an overall classification accuracy of 99.2%.

The arrow-road markings on a road surface typically become illegible or unidentifiable as the car tires erode the paint on the marking. Although this makes it difficult to correctly recognize the arrow-road marking, and represents an important problem but there is little research activity in the recognition of this type of damage to arrow-road markings. We propose to fill this research void and introduce a method that uses a convolutional neural network (CNN) to recognize six types of road markings of arrows, including damaged arrow-markings on the road surface. Recently, deep learning-based methods such as deep neural networks and CNNs have shown encouraging results in the field of computer vision and pattern recognition. Convolution can allow image-recognition networks to function in a manner similar to biological systems and produce more accurate results [20]. In recent works, a CNN has also been used for detection and classification of traffic signs [21], lane detection [22], and lane position estimation [23]. However, there is no previous research documenting studies of arrow-road marking recognition based on a CNN.

Hence, we propose a method based on a CNN to recognize damaged arrow-road markings painted on the road. The CNN-based method is a new CNN application for the recognition of the painted arrow-road marking. Our system will also provide useful results that are not affected by partial occlusions, perspective distortion, or shadow or lighting changes. We expect that this method will provide good results in conditions of poor visibility and other conditions that may inhibit collecting good-to-excellent images of the environment. Compared to the state of the art, our research is innovative in the following three ways.

- We propose a CNN-based method to recognize painted arrow-road markings. This method is new as it is not reported in the state of the art. Our method results in high accuracy of recognition and it is robust to the image quality of arrow-road marking.
- Our method is capable of recognizing severely damaged arrow-road markings. It also demonstrates good recognition accuracy in a variety of lighting conditions, such as shadowed, dark and dim arrow-road marking images that are not easily recognized.
- We used six datasets (Road marking dataset, KITTI dataset, Málaga dataset 2009, Málaga urban dataset, Naver street view dataset, and Road/Lane detection evaluation 2013 dataset) for CNN training and testing. These datasets were obtained from different countries, each with a diverse environment. The arrow-road markings of each dataset have different sizes and different image qualities. Through the intensive training of a CNN using these datasets, our method demonstrates robust performance that is independent of the nature of the datasets.

The comparisons of previous and proposed research related to road marking recognition are presented in Table 1.

The remainder of this paper is organized as follows. In Section 3, our proposed system and methodology are introduced. The experimental setup and results are presented in Section 4. Section 5 includes both our conclusions and discussions on some ideas for future work.

## 3. Proposed Method for the Recognition of Arrow-Road Markings

### 3.1. Overall Flowchart of Proposed Method

**Figure 1:**
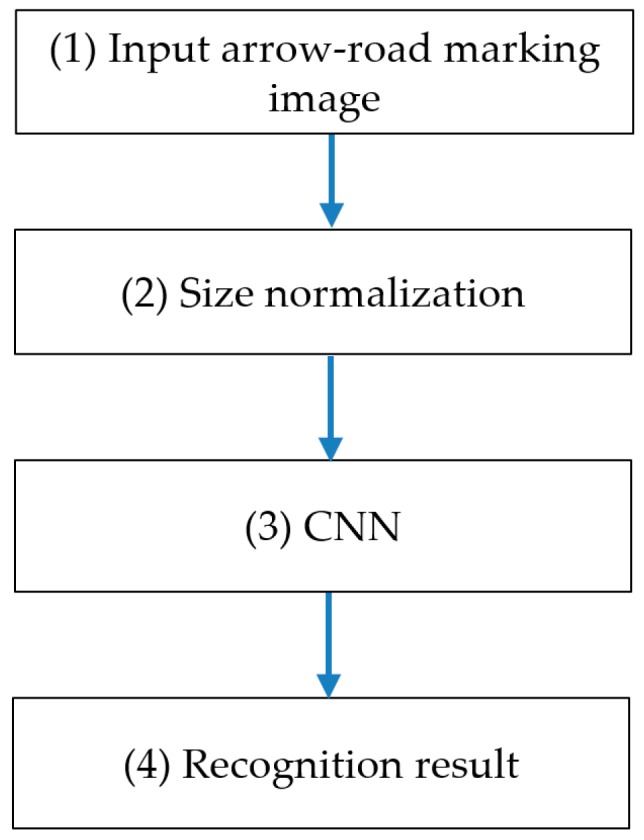
Overall flowchart of proposed method.

In Figure 1, we show the overall flowchart of our method. As the 1st step, an arrow-road marking image is inputted, and its size is normalized into the image of 265 × 137 pixels in height and width, respectively (Step (2) in Figure 1). This is because the size of input image to CNN should be same. Then, the normalized image is used as input to pre-trained CNN, and based on the output of CNN, the input arrow-road marking image is determined as one of six arrow-road markings. In our research, six types of arrow-road markings are recognized, such as forward arrow (FA), forward-left arrow (FLA), forward-left-right arrow (FLRA), forward-right arrow (FRA), left arrow (LA), and right arrow (RA). Detailed explanations of CNN structure are shown in Section 3.2, Section 3.3 and Section 3.4.

### 3.2. Architecture of the CNN

**Figure 2:**
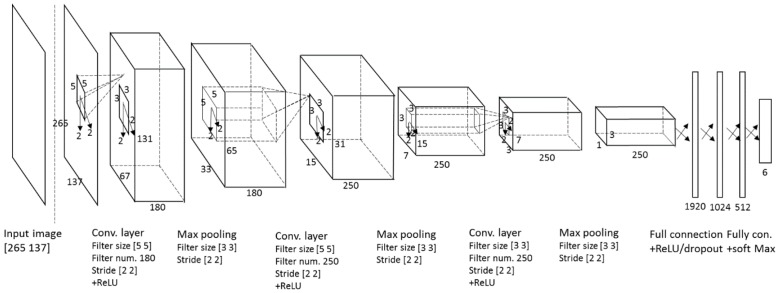
Convolutional neural network (CNN) architecture.

In our research, we introduce an arrow-road marking recognition method based on a CNN [25]. The entire process of our CNN architecture is shown in Table 2 and Figure 2. The network consists of the following specific layers: (1) three convolutional layers, each with: (2) a rectified linear unit (ReLU) layer; (3) a cross channel normalization (CCN) layer; and (4) a max pooling layer. A heap of convolutional layers is followed by four fully connected layers. Each fully connected layer is followed by a ReLU layer for the first three fully connected layers. A dropout layer is inserted before the fourth fully connected layer, which is then followed by a softmax layer and a classification layer. In the following subsections, we describe each of these layers in detail.

### 3.3. Feature Extraction by Three Convolutional Layers

We use gray-scale images as the input whose height and width are 265 and 137 pixels, respectively. Therefore, the first convolutional layer requires 265 × 137 × 1, and it is convolved with 180 filters having size 5 × 5 × 1 at stride two. In this case, the number of weights per filter is 5 × 5 × 1 = 25, and the total number of parameters in the convolution layer is (25 + 1) × 180 = 4680, such that 1 represents the bias. The size of the feature map is 131 × 67 × 180 in the first convolutional layer, such that 131 and 67 are the output height and width, respectively, calculated based on (output height (or width) = (input height (or width) − filter height (or width) + 2 × padding)/stride +1 [26]). The outputs pass through the ReLU layer and a cross channel normalization layer. After processing by the max pooling layer with filters of size 3 × 3 applied with a stride of two, every depth slice in the input is down-sampled by two along height and width. Therefore, the output of the max pooling layer is calculated as 65 ((131 − 3 + 2 × 0)/2 + 1) × 33 ((67 − 3 + 2 × 0)/2 + 1) × 180, based on the equation (output height (or width) = (input height (or width) − filter height (or width) + 2 × padding)/stride +1 [26]). The depth dimension remains unchanged at 180 after the max pooling operation. The size of the feature map after the max pooling layer is 65 × 33 × 180, and this is convolved with the second convolutional layer that uses 250 filters, each having size 5 × 5. This is followed by the max pooling layer with filters of size 3 × 3, applied with a stride of two that reduces the size of the feature map. Then, the third convolutional layer also has 250 filters, each having size 3 × 3 at stride two. After applying another max pooling layer with filters having size 3 × 3 with a stride of two pixels, the output is represented by 750 (=3 × 250) feature maps, which are used as the inputs to the first fully connected layer.

### 3.4. Classification by Four Fully Connected Layers

Our CNN structure consists of four fully connected layers. The first fully-connected layer has 750 and 1920 nodes for input and outputs, respectively. The output values pass through a ReLU layer. The second fully connected layer has 1920 and 1024 nodes for input and outputs, respectively. The third fully connected layer has 1024 and 512 nodes for input and outputs, respectively. The dropout technique is applied before the fourth fully connected layer [20,25,27], which randomly sets to zero the output of each hidden node based on a predetermined probability. In our research, we used the optimal probability of 0.65 obtained from experiments. Then, the fourth fully connected layer has 512 and 6 nodes for input and outputs, respectively. Through the softmax function [25], the final output can be obtained.

## 4. Experimental Results

### 4.1. Experimental Data and Environment

**Figure 3:**
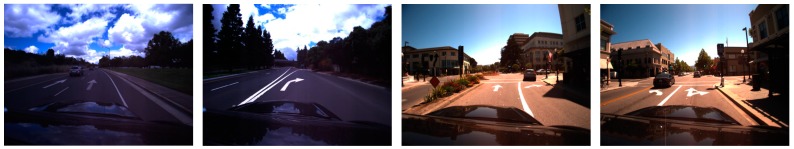
Example images from Road marking dataset.

**Figure 4:**
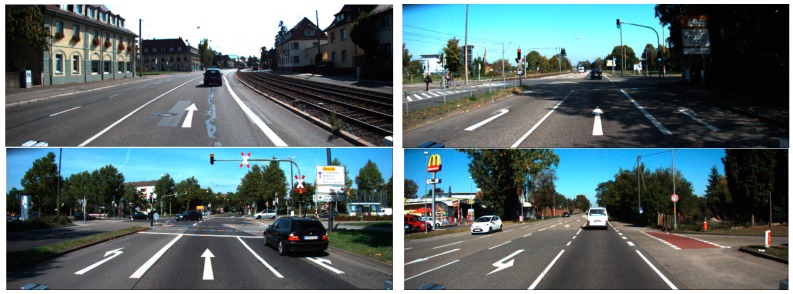
Example images from Karlsruhe institute of technology and Toyota technological institute at Chicago (KITTI) dataset.

**Figure 5:**
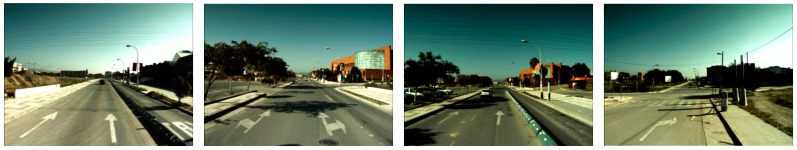
Example images from Málaga dataset 2009.

**Figure 6:**
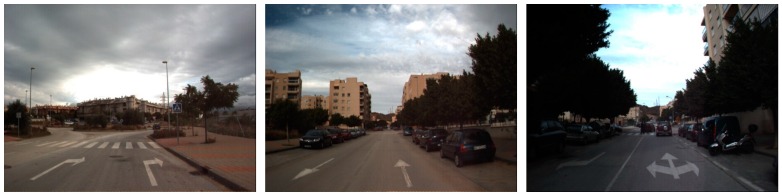
Example images from Málaga urban dataset.

**Figure 7:**
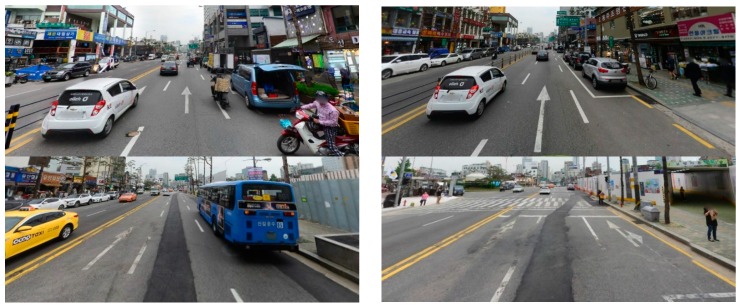
Example images from Naver street view dataset.

**Figure 8:**
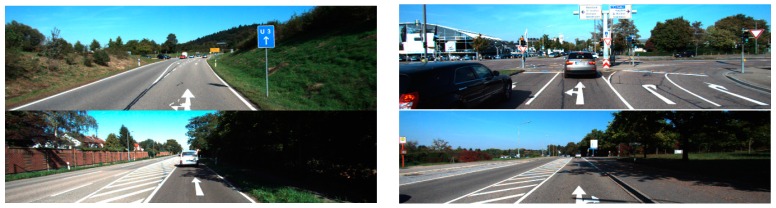
Example images from Road/Lane detection evaluation 2013 dataset.

As shown in Figure 3, Figure 4, Figure 5, Figure 6, Figure 7 and Figure 8, we used the Road marking dataset [6,28], KITTI dataset [29,30], the Málaga dataset 2009 [31], the Málaga urban dataset [32], the Naver street view dataset [33], and the Road/Lane detection evaluation 2013 dataset [34] to generate our training and testing data. Each dataset has different image sizes with various illumination and shadow attributes. Six types of arrow-road markings were obtained from the images of these datasets. They are classified into six categories: FA, FLA, FLRA, FRA, LA, and RA.

**Figure 9:**
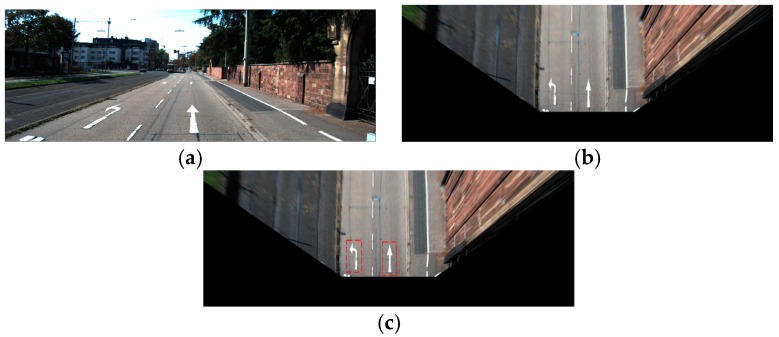
Obtaining the inverse perspective mapping (IPM) image and arrow markings: (**a**) original image; (**b**) IPM transformation; and (**c**) the obtained arrow markings.

The datasets were created from different cameras positioned to capture various orientations and perspectives. Therefore, we first applied an inverse perspective mapping (IPM) transform [35] to convert the images to IPM, from which the arrow-markings were obtained. Since each dataset requires different information regarding camera position and orientation, different geometric parameters were used for each dataset when converting the images to IPM images. The perspective transformation matrix was calculated for each dataset, and the IPM image was obtained as shown in Figure 9b. Then, arrow-markings were obtained from the images as shown in Figure 9c.

**Figure 10:**
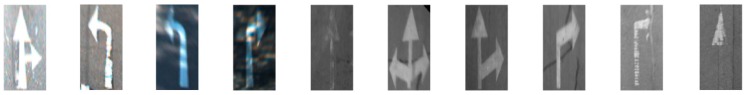
Examples of arrow markings after size normalization and bi-linear interpolation.

We can see the examples of arrow markings in Figure 10. In order to use the training and testing of our CNN, we set the size of all of the arrow marking images to 194 × 94 pixels in height and width, respectively, by size normalization and bi-linear interpolation. As shown in Figure 10, the arrow markings from six datasets including various illumination change, shadow, and severe damage, were used for training and testing of the CNN.

Previous CNN research indicated that a large number of training datasets plays a very important role in enhancing the recognition performance of the CNN [27]. Given this knowledge, we performed data augmentation from the original arrow marking images by executing shifting and mirroring operations on the region of the arrow marking in order to obtain a large amount of data. Using these results, we increased the data size by a factor of 98, and 163,809 arrow-road marking images were obtained, as shown in Table 3. The numbers of each arrow marking image are also shown in Table 3.

Training and testing were performed on a desktop computer configured with an Intel^®^ Core™ i7-6700 CPU @ 3.40 GHz (4 CPUs) [36], memory of 64 GB, and Graphics card of NVIDIA GeForce GTX TITAN X (3072 CUDA cores) with memory of 12 GB (NVIDIA, Santa Clara, CA, USA) [37].

### 4.2. Training

**Figure 11:**
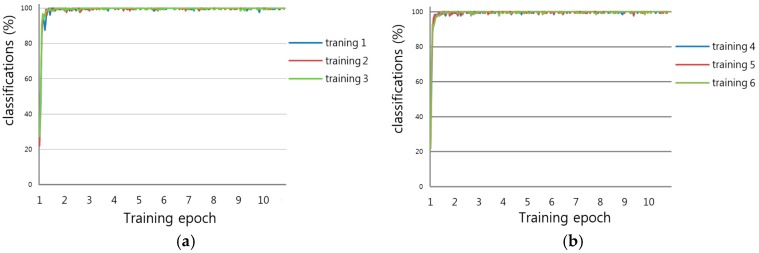

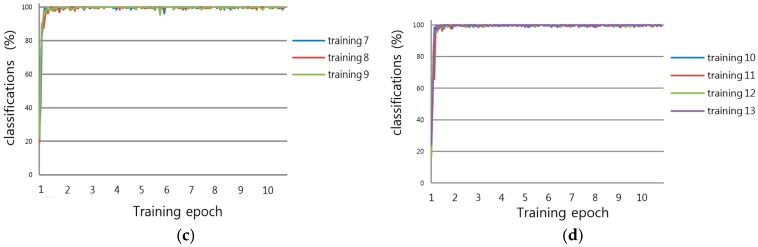
Classification accuracies of training data over 13-fold cross validation; “classifications” means “classification accuracy of training data”: (**a**) Training 1–3; (**b**) Training 4–6; (**c**) Training 7–9; (**d**) Training 10–13.

We applied a MATLAB implementation [38] to train the CNN model. Through bi-linear interpolation, the experimental image, with a size of 194 × 94 pixels, was resized to 265 × 137 pixels of 8 bit gray, and then used for the CNN training and testing. For experiments, we performed a 13-fold cross validation. That is, among the entire dataset of 163,809 images, 92.7% (151,809 images) and 7.3% (12,000 images) were randomly selected for training and testing, respectively, and thirteen iterations of this procedure were executed. Using these results, we measured the average accuracy of recognition of an arrow marking. Our training process iterated over 10 epochs, performing 1200 iterations for each epoch. The initial learning rate was 0.01 with a learn-rate-drop factor of 0.1 after every 25 epochs. The classification accuracies of the training data, according to the training epoch when performing the 13-fold cross validation, are shown in Figure 11. Figure 11 also shows that the classification accuracy of 100% was obtained in each training case of the 13-fold cross validation.

**Figure 12:**
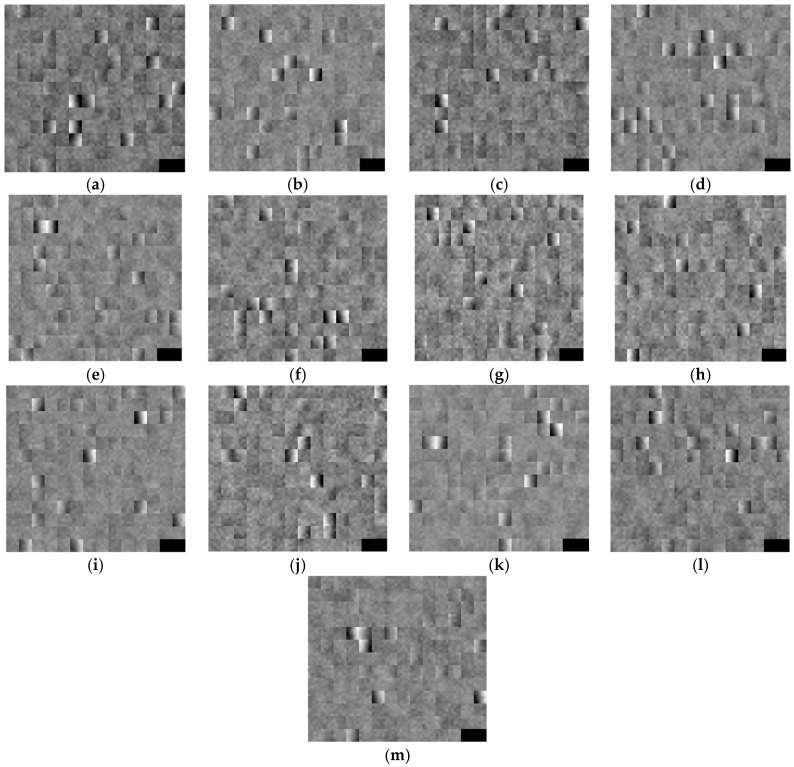
The obtained filters from the 1st convolution layer through training: (**a**–**m**) the filters from the 1st–13th trainings among 13-fold cross validation are presented, respectively.

In Figure 12, we show the obtained filters from the 1st convolution layer through training. As shown in the Table 2, the size of each filter is 5 × 5 and the number of filter is 180. For higher visibility, each filter is increased into 25 × 25 pixels by bi-linear interpolation. Because the number of filter is 180, the last two (right-bottom) squares among 182 (=14 × 13) squares do not present the obtained filters and they are shown as black color.

### 4.3. Testing: Measuring the Accuracies of Arrow-Road Marking Recognition

With the trained CNN and testing data, we measured the accuracies of recognition of arrow-road marking. Table 4 shows the summated confusion matrix of tests 1–13. The results showed that the classification results are consistent and highly accurate for arrow-road marking recognition. In the case of LA, the recognition failure rate was higher than that of other classes. Most of these failed LA cases were recognized as a RA.

We then measured the accuracies of the arrow marking recognition using Equations (1)–(4) [39]. #TN, #TP, #FN and #FP represent, respectively, the number of true negatives (TNs), true positives (TPs), false negatives (FNs) and false positives (FPs). TN represents the case such that the arrow marking excluded in the input image is correctly unrecognized, whereas TP represents the case such that the arrow marking in the input image is correctly recognized. FN represents the case such that the arrow marking in the input image is incorrectly unrecognized, whereas FP is the case where the arrow marking excluded in the input image is incorrectly recognized. The minimum and maximum values of PPV, TPR, ACC, and F_score are 0% and 100%, respectively, such that 0% and 100% represent the lowest and highest accuracies, respectively. As shown in Table 5, our method can correctly recognize the arrow markings from various datasets including illumination changes, and shadow and damage at an accuracy rate higher than 99.8%.

(1) $$\mathrm{Positive}\;\mathrm{predictive}\;\mathrm{value}\;\left(\mathrm{PPV}\right)=\frac{\#\mathrm{TP}}{\#\mathrm{TP}+\#\mathrm{FP}}$$

(2) $$\mathrm{True}\;\mathrm{positive}\;\mathrm{rate}\;\left(\mathrm{TPR}\right)=\frac{\#\mathrm{TP}}{\#\mathrm{TP}+\#\mathrm{FN}}$$

(3) $$\mathrm{Accuracy}\;\left(\mathrm{ACC}\right)=\frac{\#\mathrm{TP}+\#\mathrm{TN}}{\#\mathrm{TP}+\#\mathrm{TN}+\#\mathrm{FP}+\#\mathrm{FN}}$$

(4) $$F\_\mathrm{score}=2\times \frac{\mathrm{PPV}\;\times \;\mathrm{TPR}}{\mathrm{PPV}\;+\;\mathrm{TPR}}$$

In the next experiment, we compared the accuracies of our method with those of the previous method [40]. As shown in Table 6, our method outperforms the previous method.

**Figure 13:**
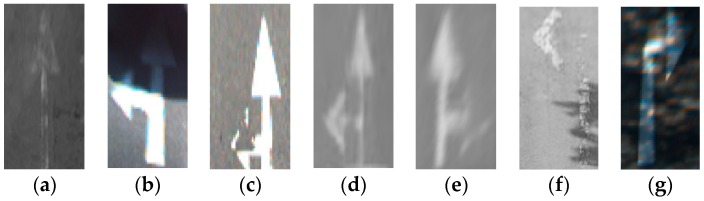
Examples of correct recognition cases: (**a**) forward arrow (FA); (**b**–**d**) forward-left arrow (FLA); (**e**) forward-right arrow (FRA); (**f**) left arrow (LA); (**g**) right arrow (RA).

**Figure 14:**
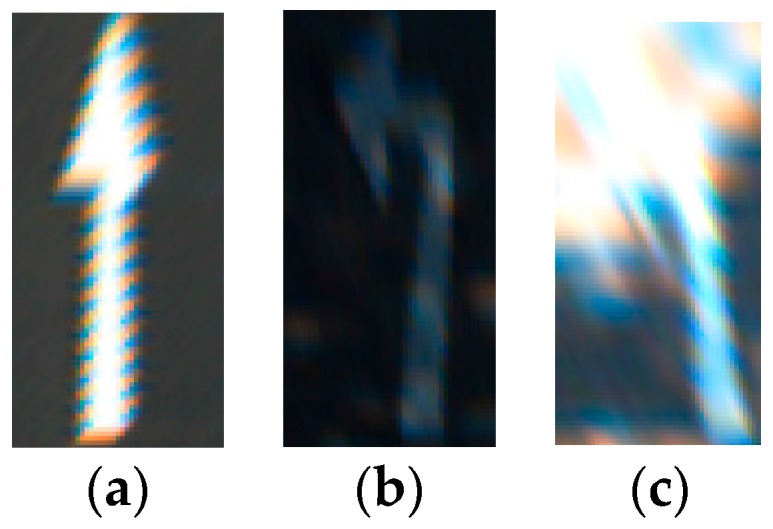
Examples of incorrect recognition cases: (**a**) FA is incorrectly recognized into FLA; and (**b**,**c**) LA is incorrectly recognized into RA.

In Figure 13, we show the examples of correct recognition cases, which show that our method can correctly recognize the arrow markings even in the case of being severely damaged. In addition, the examples of incorrect recognition cases are shown in Figure 14. As shown in Figure 14, most of the incorrect recognition is due to the low image quality by image blurring, severe shadow and bright sunlight.

Other reason of the confusions is as follows. The largest confusions occur between LA (left arrow) and RA (right arrow) (210 samples of LA are incorrectly recognized into RA as shown in Table 4). This is caused by the structure of our CNN of Table 2 and Figure 2. As shown in Table 2, through the 1st–3rd convolutional layer, the size of feature maps is reduced (from 265 (height) × 137 (width) pixels to 3 (height) × 1 (width) pixels), which means that the height and width of one feature map become 3 and 1 pixels, respectively, in the max pooling layer of the 3rd convolutional layer. Because the width of feature map is just 1 pixel, the LA and RA show similar patterns each other in this feature map, which increases the largest confusions between LA and RA. Same cases happen between FLRA and FA in addition to FRA and LA as shown in Table 4.

To solve this problem, we revised our CNN structure to obtain 3 (height) × 2 (width) pixels in the max pooling layer of the 3rd convolutional layer of Table 2. Because the width of feature map is 2 pixels, the LA and RA (FLRA and FA in addition to FRA and LA) can show different patterns from each other in this feature map. As shown in Table 7, the confusions between LA and RA by our revised CNN are greatly reduced from 210 to 13. In addition, the confusions between other arrow-road markings are removed. Consequently, average ACC and F_score by revised CNN are higher than those by original CNN as shown in Table 6 and Table 8.

## 5. Conclusions

In this research, we proposed a method to recognize damaged arrow markings residing on a road. We deployed a CNN and collected training and testing data from various types of datasets. We trained the CNN to recognize arrow markings in the presence of varying illumination, and shadow and damage conditions. A simple CNN was designed and then trained using various “raw” datasets, i.e., the datasets were not preprocessed for noise removal, contrast normalization, or brightness correction. The experimental results demonstrate that the accuracy of recognizing arrow markings by the proposed method was consistently higher and more reliable, relative to the previous method.

In the future, we plan to implement and integrate our method on an actual automobile, and measure the performance while driving the car. We will then compare the accuracy of our method with the accuracies of other known CNN implementations.

## Figures and Tables

**Table 1:** Comparisons of previous and proposed methods.

Category	Methods	Advantages	Disadvantages	Performance	Year	Ref.
Non-learning-based	Geometric parameter optimization	Fast processing speed	The number of thresholds to be set is large	True positive rate (TPR) of 90% and 78% for crosswalks and arrows, respectively	2011	[5]
	HOG features and template matching	A good ability to cope with the cases of limited occlusions and the variations of lighting condition	- In bright conditions, system is sensitive to shadows - High FPR for simple but important signs such as forward arrows	TPR of 90.1% and false positive rate (FPR) 0.9%	2012	[6]
	Template matching	Fast processing speed	Damaged road marking can cause misclassification, and the classification accuracy is affected by the illumination variation	Detection rate of 95.8% and 84% on the highway and city roads, respectively	2012	[7]
Learning-based	HOG features and total error rate (TER)-based classifier	Fast computing time compared to SVM-based method	Damaged or shadowed markings increase FPR	Overall classification accuracy of 99.2%	2015	[19]
	HOG features and SVM	Showing high accuracy with the trained datasets	Recognition accuracy can be affected by damaged or shadowed markings	Quantitative accuracies were not reported	2015	[8]
				F-measure of 0.91	2015	[9]
				Average accuracy of 91.7%	2014	[24]
	Fourier descriptor and KNN classifier	Robust to noises on road marking	Sensitive to occlusion, dirty markings or poor visibility	Average error of 6%	2004	[12]
	Artificial Neural Network	Higher accuracy with trained datasets compared to non-learning-based method	Performance of testing data can be affected by trained dataset	Quantitative accuracies were not reported	1994	[15]
				The average accuracy of white markings is about 71.5%, and for orange markings it was about 46%	2014	[16]
				Accuracy of 85% for arrows	2012	[17]
	BING, PCA network, and SVM classifier	The area of road marking can be detected by BING method without lane detection	Performance of testing data can be affected by trained dataset	Accuracy of 96.8%	2015	[18]
	**Proposed method (CNN)**	Arrow-road markings in various environments including damaged ones can be correctly recognized independent of the kinds of datasets by intensive training of CNN	Time consuming procedure for training is required for CNN	Average accuracy and F_score are 99.88% and 99.94%, respectively		

**Table 2:** The CNN architecture used in our research.

Layer Type	Number of Filters	Size of Feature Map	Size of Kernel	Number of Stride
Image input layer		265 (height) × 137 (width) × 1 (channel)		
1st convolutional layer	180	131 × 67 × 180	[5 5]	[2 2]
ReLU layer				
CCN layer				
Max pooling layer	180	65 × 33 × 180	[3 3]	[2 2]
2nd convolutional layer	250	31 × 15 × 250	[5 5]	[2 2]
ReLU layer				
CCN layer				
Max pooling layer	250	15 × 7 × 250	[3 3]	[2 2]
3rd convolutional layer	250	7 × 3 × 250	[3 3]	[2 2]
ReLU layer				
CCN layer				
Max pooling layer	250	3 × 1 × 250	[3 3]	[2 2]
1st fully connected layer		1920		
ReLu layer				
2nd fully connected layer		1024		
ReLu layer				
3rd fully connected layer		512		
ReLu layer				
Dropout layer				
4th fully connected layer		6		
Softmax layer				
Classification layer (output layer)				

**Table 3:** Number of data for our experiments.

	FA	FLA	FLRA	FRA	LA	RA	Total
Number of data	32,686	17,885	22,344	17,885	36,766	36,243	163,809

**Table 4:** The summated confusion matrix of tests 1–13.

Total of Testing 1–13		Recognized Arrows					
		FA	FLA	FLRA	FRA	LA	RA
Actual arrows	FA	31,447	1	0	1	0	2
	FLA	0	18,101	0	1	0	0
	FLRA	21	0	22,254	0	0	0
	FRA	0	0	0	18,824	24	0
	LA	1	0	0	0	33,334	210
	RA	0	0	0	0	0	31,779

**Table 5:** Accuracies of arrow marking recognition by our method (unit: %).

# of Testing		FA	FLA	FLRA	FRA	LA	RA
Testing 1	ACC	100	100	100	100	99.45	100
	F_score	100	100	100	100	99.72	100
Testing 2	ACC	100	100	100	100	99.44	100
	F_score	100	100	100	100	99.72	100
Testing 3	ACC	99.91	100	100	100	99.05	100
	F_score	99.96	100	100	100	99.52	100
Testing 4	ACC	100	100	100	100	99.02	100
	F_score	100	100	100	100	99.51	100
Testing 5	ACC	100	100	100	100	99.12	100
	F_score	100	100	100	100	99.56	100
Testing 6	ACC	100	100	100	100	99.41	100
	F_score	100	100	100	100	99.70	100
Testing 7	ACC	99.96	100	100	100	99.34	100
	F_score	99.98	100	100	100	99.67	100
Testing 8	ACC	100	100	100	99.11	100	100
	F_score	100	100	100	99.55	100	100
Testing 9	ACC	100	100	100	100	99.44	100
	F_score	100	100	100	100	99.72	100
Testing 10	ACC	99.96	100	100	100	99.48	100
	F_score	99.98	100	100	100	99.74	100
Testing 11	ACC	100	100	99.22	100	100	100
	F_score	100	100	99.61	100	100	100
Testing 12	ACC	100	100	100	100	99.44	100
	F_score	100	100	100	100	99.72	100
Testing 13	ACC	100	99.93	100	100	98.99	100
	F_score	100	99.96	100	100	99.49	100
Average ACC		99.99	99.99	99.94	99.93	99.40	100
		**99.88**					
Average F_score		99.99	99.997	99.97	99.97	99.70	100
		**99.94**					

**Table 6:** Comparisons of accuracies of recognition of arrow marking by our method with previous method (unit: %).

	Our Method	Previous Method [40]
Average ACC	99.88	92.8
Average F_score	99.94	93.9

**Table 7:** The summated confusion matrix by revised CNN.

Total of Testing 1–13		Recognized Arrows					
		FA	FLA	FLRA	FRA	LA	RA
Actual arrows	FA	31,451	0	0	0	0	0
	FLA	0	18,102	0	0	0	0
	FLRA	0	0	22,275	0	0	0
	FRA	0	0	0	18,848	0	0
	LA	0	0	0	0	33,532	13
	RA	0	0	0	0	0	31,779

**Table 8:** Accuracies of recognition of arrow marking by revised CNN (unit: %).

	Our Method
Average ACC	99.99
Average F_score	99.99
